# Lack of knowledge about sexually transmitted infections among women in North rural Vietnam

**DOI:** 10.1186/1471-2334-9-85

**Published:** 2009-06-06

**Authors:** Pham Thi Lan, Cecilia Stålsby Lundborg, Ingrid Mogren, Ho Dang Phuc, Nguyen Thi Kim Chuc

**Affiliations:** 1 Division of Global Health (IHCAR), Department of Public Health Sciences, Karolinska Institutet, Stockholm, Sweden; 2 Department of Dermato-Venereology, Hanoi Medical University, Hanoi, Vietnam; 3 The Nordic School of Public Health and Apoteket AB, Göteborg, Sweden; 4 Department of Clinical Sciences, Obstetrics and Gynaecology, Department of Public Health and Clinical Medicine, Epidemiology and Public Health Sciences, Umeå University, Umeå, Sweden; 5 Department of Probability and Mathematical Statistics, Institute of Mathematics, Hanoi, Vietnam; 6 Department of Public Health, Hanoi Medical University, Hanoi, Vietnam

## Abstract

**Background:**

The serious long-term complications of sexually transmitted infections (STI) in women and newborns are well-documented. Particularly, STI imply considerable social consequences for women. Low STI knowledge has been shown to be associated with unsafe sex. In Vietnam, misconceptions regarding STI exist, and rural women delay seeking care for STI. The aim of the study was to investigate knowledge of STI among women aged 15 to 49 years in a rural district of Vietnam and to evaluate possible associations between socioeconomic factors and STI knowledge.

**Methods:**

A cross-sectional population-based study using face-to-face interviews was carried out between March and May 2006 in a demographic surveillance site in rural Vietnam. In total, 1805 women aged 15–49 years were randomly selected to participate in the study. The interviews were based on a structured questionnaire including questions on sociodemographic characteristics of the women and their knowledge about STI. Each correct answer was scored 1, incorrect or do not know answer was scored 0. Multivariate analyses were applied to examine associations between socio-economic conditions and STI knowledge. Intra-cluster correlation was calculated to examine similarities of STI knowledge within clusters.

**Results:**

Of the 1,805 respondents, 78% (73% married vs. 93% unmarried, p < 0.001) did not know any symptoms of STI, 50% could not identify any cause of STI, 59% (54% married vs. 76% unmarried, p < 0.001) did not know that STI can be prevented. Only 31% of the respondents (36% married vs. 14% unmarried, p < 0.001) answered that condom use could protect against STI, and 56% considered partner treatment necessary. Of 40 possible correct answers, the mean knowledge score was 6.5 (range 0–26, median 6). Young, unmarried women and women who lived in the highlands or mountainous areas demonstrated very low levels of STI knowledge (regression coefficients -1.3 and -2.5, respectively, p < 0.001). Experience of an induced abortion was significantly associated with a higher level of knowledge.

**Conclusion:**

The low levels of STI knowledge found among women of reproductive age in a rural district of Vietnam indicate an urgent need of health education interventions, of which, young and unmarried women should be specifically targeted.

## Background

Sexually transmitted infections (STI) constitute a huge health and economic burden for low-income countries [1]. The presence of an STI, particularly an ulcer-causing STI, can enhance the acquisition and transmission of HIV [2]. The serious long-term complications of STI in women and newborns are well-documented [3-5]. Furthermore, STI imply considerable social consequences particularly for women, including stigmatisation, domestic abuse and even abandonment. In low-income countries, women are more vulnerable to reproductive health problems with serious sequelae of STI, and less likely to receive appropriate and timely care than women in high-income countries [3]. Low STI knowledge has been shown to be connected with unsafe sex practices and HIV [6]. In low-income countries, STI often go undiagnosed and untreated due to lack of knowledge and/or non-availability of healthcare facilities. Little emphasis on educational and other efforts to prevent infection occurring in the first place is one of common reasons why STI control programmes often fail in low-income countries [1].

In Vietnam, the potential for significant HIV and STI epidemics has been documented [7]. Annually, more than 130,000 STI cases are reported nationwide [report of the National Institute of Dermato-Venereology, Vietnam, 2007], which probably represent an underreporting of the true situation because of self-medication [8], treatment by drug sellers [9], and non-reported STI cases from private providers. Furthermore, there is a rapid increase in new HIV cases, of which the majority are believed to have been sexually transmitted [10]. STI is common among high-risk groups [11-13] and also not uncommon in the general population [14]. Urbanisation and social transition in Vietnam have resulted in labour migration, which can lead to unsafe sexual behaviour including increasing STI/HIV risks [5]. The existence of misconceptions about STI among people in the community [15] as well as delay in seeking care for STI among women in rural areas [16] have been shown recently. Data on knowledge about STI in Vietnam are limited [17]. Most studies of STI-related knowledge are based on purposive samples [15,18,19], at STI treatment facility [16], or have focused on high-risk groups [20]. Understanding people's knowledge regarding STI could provide an important basis for the development of interventions to promote early healthcare-seeking behaviour and protective practice for STI, and avoid its complications.

The aim of this study was to investigate knowledge of STI among women aged 15–49 in rural Vietnam and, further to evaluate possible associations between socioeconomic factors and STI knowledge.

## Methods

### Study site and population

The study was conducted within a demographic surveillance site (named FilaBavi) in Bavi district, northern Vietnam. The district is located 60 km west of Hanoi, the capital and covers an area of 410 km^2^, including lowland, highland, and mountainous areas. The district was considered typical of Vietnam in socioeconomic and health status [21]. The number of inhabitants is approximately 240,000 people, living in 32 communes. Each commune has 6,000 to 10,000 inhabitants divided into a number of villages. Farming and livestock breeding are the main economic activities in the district.

The FilaBavi was developed within the Health Systems Research Project supported by Sida/SAREC, Sweden, with the overall aim was to implement a longitudinal epidemiological surveillance system that could generate basic health and healthcare data, supply information for health planning, serve as a background and sampling frame for specific studies, and constitute a setting for epidemiological training. Sixty nine clusters (out of 352) in the district were randomly selected with probability proportional to population size to constitute the sample for FilaBavi. The FilaBavi study base has 69 clusters with approximately 12,000 households and 51,000 inhabitants (approximately 20% of the district's total population). Women aged 15 to 49 years constitute 28% of the population. Of them, approximately 70% are married. In FilaBavi, a cluster was defined as a village. On average, there were about 160 households and 670 inhabitants in each cluster.

The current study was a cross-sectional study using a face-to-face interview based on a structured questionnaire. This was part of a larger project in the same setting that comprises a study on prevalence of reproductive tract infections (RTI) including STI [14]. The study sample size was estimated in order to investigate RTI/STI prevalence among married women in the primary study [14]. We used random sampling method to select 17 clusters (out of 69) of FilaBavi for the study. Based on the lists of women, we selected randomly about 100 to 110 women aged 15 to 49 years in each cluster for the interview. Totally, 1,805 women were selected to participate in the current study.

### Questionnaire development

The questionnaire (see Additional file 1) was developed mainly based on findings of a qualitative study conducted in the same setting [15] and previous studies elsewhere [19,22]. It was reviewed with the surveyors of FilaBavi, pre-tested and revised several times to ensure if it is understandable and suitable to the sociocultural context of the study area. The questionnaire was pilot tested in one cluster (100 women), which was not included in the analysis for this paper. The questionnaire contained questions regarding: respondents' socio-demographic information; experiences related to childbearing; and questions concerning STI knowledge including characteristics of abnormal vaginal discharge, suspected symptoms, possible causes, transmission, curability, complications, partner treatment and prevention. Information on self-reported STI or STI-related symptoms during life-time and self-reported STI-related symptoms during the past 6 months and at the time of the interview was also requested.

### Data collection and analysis

The study was conducted between March and May, 2006. Firstly, the surveyors and the field supervisors of FilaBavi, who lived in the district, were trained to be familiar with the questionnaire and were informed about data collection procedures. Then the interview was performed privately at respondents' homes by 42 trained female surveyors. The field supervisors checked all the collected forms and conducted re-interviews of approximately five percent of the forms to ensure the quality of data collected. Data regarding the economic status of the women were extracted from the available demographic database of FilaBavi.

Collected data were processed and analysed using SPSS version 13. Overall STI knowledge was evaluated by scoring correct answers. All correct responses were given a score of 1 and incorrect or "do not know" responses were given a score of 0. Proportions, mean, median, minimum and maximum were used for the descriptive analysis. Chi-Square tests were performed to examine the difference between proportions. Linear regression models were employed to explain relations between dependent and independent variables controlling for confounders. Multivariate logistic regression was used to identify factors influencing women's STI knowledge. Moreover, MLwiN version 1.1 was used for multilevel linear regression analysis and for calculation of intra-cluster correlation coefficient (ICC) examining the similarities of knowledge of women within clusters. Interviewer variances were also calculated. Regression coefficients were adjusted for ICC.

### Ethics

The study was approved by the regional ethics committee in Stockholm, Sweden and by Hanoi Medical University, Vietnam. The study's aims were explained and verbal consent was granted by all respondents before interview. The respondents were informed that the participation was voluntary, and that they could withdraw at any time of the study without any adverse consequence. They were encouraged to tell their experiences and views of the issues under study. They were also assured that they would remain anonymous in any future written report from the study, and their responses would be treated with confidentiality.

## Results

### The characteristics of respondents

We had a response rate of 100%. Among the 1,805 respondents, 1,360 were married (including married and lived with husband 1,295, married but separated 8, divorced 22, or widowed 35) and 445 were unmarried, the mean age was 32 years. Almost all women aged 15 to 19 years (97%) were unmarried. Among married women, the mean age at first marriage was 20.9 years (range 13–43) and the mean number of children was 2.3 (range 0–8). Nearly half of the respondents lived in the lowland. Majority of the respondents were farmers. Literacy reached 99.6% in the study population. The characteristics of respondents are shown in Table 1.

**Table 1:** Socio-demographic characteristics and childbearing experience of 1805 women aged 15–49 years in North rural Vietnam

	Unmarried	Married	Total
	% (n = 445)	% (n = 1360)	% (N = 1805)
**Age group (years)**			
15 – 19	61.6	0.6	15.6
20 – 29	31.5	24.1	25.9
30 – 39	3.8	35.4	27.6
40 – 49	3.1	39.9	30.9
**Education**			
Illiterate	0.9	0.3	0.4
Primary school (< 6 years)	9.4	14.8	13.4
Secondary school (6–9 years)	29.2	65.5	56.6
High school (10–12 years)	49.0	12.9	21.8
College/university	11.5	6.5	7.8
**Occupation**			
Farmer	27.6	78.6	66.0
Government staff	1.3	6.4	5.1
Worker/hire labour	11.7	5.0	6.6
Trader and others (housewife, unemployed...)	4.9	9.9	8.7
Student ^±^	54.4	0.1	13.5
**Economic status **^#^			
1^st ^quintile group	12.6	13.5	13.3
2^nd ^quintile group	21.1	18.8	19.4
3^rd ^quintile group^c^	19.6	20.1	20.0
4^th ^quintile group	19.8	22.3	21.7
5^th ^quintile group	27.0	25.2	25.6
**Place of residence**			
Lowland	42.0	45.1	44.3
Highland	31.0	29.6	30.0
Mountainous	27.0	25.3	25.7
**Experience of childbirth**	NA^¥^	97.6	-
**Had at least one induced abortion**	NA^¥^	37.4	-
**Had at least one adverse pregnancy outcome**^§^	NA^¥^	20.5	-

^NA ^Not applicable, these questions were not asked because of the sensitiveness

^± ^Secondary school 18%, high school 69%, college/university 13%, under 21 years of age 92%

^# ^Based on household's wealth index performed as principal component from economic indicators (including incomes, expenditures and debt), housing and sanitary conditions, land area and household assets

^¥ ^Being unmarried but having experience of childbirth: 7, none of them reported having induced abortion or adverse pregnancy outcome

^§ ^Miscarriage 216, still birth 38, premature birth 27, and neonatal death 10

### Knowledge about STI

#### Overall STI knowledge

Table 2 displays the proportions of unmarried and married women answering questions regarding STI knowledge. The most common signs of abnormal vaginal discharge, defined by the respondents, were odour and excessive amount (35.5% and 24.8% of responses, respectively). Three-fourths of unmarried women did not know the common characteristics of abnormal vaginal discharge. Among suspected symptoms of STI, vaginal itching was the most frequently mentioned by the respondents (16%), followed by abnormal vaginal discharge (9.5%). Only 1.3% women considered urethral discharge in men as a suspected symptom of STI. Similarly, low abdominal pain (in women), and dyspareunia or dysuria was rarely mentioned by the respondents. Seventy eight percent of women (73% married vs. 93% unmarried, p < 0.001) did not report knowledge of any symptom of STI. Only five percent of the women knew that possible causes of STI were micro organisms. Bad hygiene and having sex during menstruation or soon after delivery were mentioned as "causes" (based on the respondents' own words) of STI by 11.5% and 2.8% of women, respectively. Two-fifths of the women answered that STI was caused by being unfaithful or having unsafe sex. Half of the respondents did not know any "cause" of STI. The proportions of respondents who correctly answered the question concerning STI transmissibility and the necessity of partner treatment were 76.5% and 55.9%, respectively. Concerning STI curability, 16.3% women considered gonorrhoea and/or syphilis as curable diseases. Twenty one percent of the respondents mentioned HIV/AIDS as incurable while 14 women thought HIV/AIDS could be cured. Concerning sequelae of untreated STI, the correct answer rates were very low, while 59% of the women (54% married vs. 76% unmarried, p < 0.001) did not know of any complication. There were significantly more unmarried than married women who were unaware that STI could be prevented. The results also demonstrated significant differences in proportions of unmarried and married women who mentioned ways of STI prevention such as using condoms, avoiding injecting illicit drugs, and keeping good hygiene (Table 2).

**Table 2:** Responses to the questions about STI knowledge among 1805 women aged 15–49 years in North rural Vietnam

*Correctly identifying*	Unmarried % (n = 445)	Married % (n = 1360)	Total % (N = 1805)	P value^a^
**Characteristics of abnormal vaginal discharge**				
Greater amount than usual	12.1	28.9	24.8	0.000
Odour	14.4	42.4	35.5	0.000
Changes of colour (yellow-green/powdery/foamy/blood-stained)	4.7	24.0	19.2	0.000
**Suspected symptoms**				
Abnormal vaginal discharge (in women)	1.8	12.0	9.5	0.000
Urethral discharge (in men)	0.2	1.6	1.3	0.023
Genital ulcers/genital warts	2.9	9.3	7.8	0.000
Lower abdominal pain (not related to menses)	0.2	4.7	3.6	0.000
Genital itching	4.5	19.8	16.0	0.000
Dyspareunia/dysuria	0.2	5.7	4.3	0.000
**Possible "causes" of STI**				
Microorganisms (bacteria/virus)	2.2	5.8	4.9	0.003
Being unfaithful/unsafe sex	31.7	43.4	40.5	0.000
Unsafe blood transfusion/intravenous drug use	11.2	14.8	13.9	0.061
**STI can be transmitted**	62.0	81.2	76.5	0.000
**Routes of transmission**				
Sexual intercourse	56.9	78.7	73.3	0.000
Blood transfusion	14.2	11.8	12.3	0.181
Sharing needle	10.1	7.3	8.0	0.055
Mother to child	8.1	5.7	6.3	0.076
**Necessity of partner examination/treatment**	33.3	63.3	55.9	0.000
**Complications of STI if untreated**				
Infertility	14.6	20.2	18.8	0.008
Cervical cancer/ectopic pregnancy	6.1	25.9	21.0	0.000
Adverse pregnancy outcome	3.8	10.3	8.7	0.000
**STI can be prevented**	44.9	71.3	64.8	0.000
**Ways of STI prevention**				
Being faithful	28.5	33.2	32.1	0.067
Using condom	14.4	35.9	30.6	0.000
Avoid injecting illicit drugs	7.2	3.6	4.5	0.001

^a ^Chi – square statistic compares married and unmarried groups

Out of 40 possible correct answers, the mean knowledge score was 6.5 (minimum 0, maximum 26, and median 6). There was an association of STI knowledge between women within cluster (ICC = 0.25, p < 0.01). The highest mean knowledge score 9.9 (minimum 0, maximum 26) was obtained from a cluster in the lowland, and the lowest mean score 3.6 (minimum 0, maximum 12) was obtained in a cluster in the mountainous area. The calculation of interviewer variances had been made showing the ICC of 0.08, p > 0.05.

A knowledge score of zero was found among 31% unmarried and 5% married women. More than a quarter (26.7%) of the respondents who were students reported no knowledge about STI. The proportion of women with a knowledge score of zero was significantly higher among women living in the remote areas (15.2%) compared to those living in the lowland (6.6%), and among women without experience of induced abortion compared to women having had at least one abortion (14.8% vs. 2.8%).

#### Factors influencing STI knowledge

The multiple linear regression model (Table 3) describes the impact of socioeconomic conditions and self-reported STI-related symptoms (independent variables) on the overall knowledge score (dependent variable). A higher level of STI knowledge was significantly associated with being married, being aged 20 to 29 years, being a worker or a government staff, higher educational level, living in the lowland, experience of induced abortion, having symptoms during the past 6 months. Knowledge was not significantly related to childbirth experience, self-reported STI/STI-related symptoms during lifetime or at the time of interview.

**Table 3:** STI knowledge scores of 1805 women and socioeconomic factors in multiple linear regression models

	Number of participants	Mean score	Linear regression	
			
			Regression coefficient^b^	P-value
**Marital status**				
Unmarried	445	4.3	-2.4	0.000
Married	1360	7.3	0	reference
**Age (years)**				
15 – 19	282	3.9	-1.3	0.003
20 – 29	468	7.1	0	reference
30 – 39	498	6.9	-0.7	0.003
40 – 49	557	7.3	-0.3	0.092
**Education**				
Primary/illiterate	250	5.1	-1.2	0.000
Secondary	1022	6.4	0	reference
High school	393	6.2	1.0	0.000
College/university	140	10.4	3.3	0.000
**Occupation**				
Farmer	1192	6.4	0	reference
Government staff	93	11.4	1.1	0.036
Worker	120	7.0	0.9	0.002
Trader/others	157	7.4	0.5	0.165
Student	243	4.6	0.8	0.046
**Place of residence**				
Lowland	799	7.6	0	reference
Highland	541	5.7	-1.3	0.000
Mountainous	465	5.7	-2.5	0.000
**Economic status**				
1^st ^quintile group	239	5.5	-0.3	0.163
2^nd ^quintile group	350	5.8	-0.2	0.283
3^rd ^quintile group	361	6.1	0	reference
4^th ^quintile group	392	6.7	0.0	0.762
5^th ^quintile group	463	7.9	0.4	0.087
**Experience of induced abortion**				
No	1298	6.0	0	reference
Yes	507	8.0	0.7	0.001
**Experience of childbirth**				
No	464	4.5	-0.7	0.244
Yes	1327	7.3	0	reference
**Self-reported STI-related symptoms during last 6 months**				
No	1160	5.9	0	reference
Yes	645	7.6	0.6	0.002

^b ^Regression coefficient in multilevel analysis controlling for intra correlations of household and cluster

Positive regression coefficients indicate positive associations

Negative regression coefficients indicate negative associations

Multivariate logistic regression analysis (not shown) showed the impact of related factors on each aspect of STI knowledge of the study women. Notably, there were significant differences concerning STI knowledge in almost all aspects between married and unmarried women, between women living in the lowlands and in the mountainous or highland areas.

## Discussion

The most important finding in our study was the low level of basic knowledge regarding STI among the respondents. The average overall knowledge score was as low as 6.5 (of 40 possible correct answers). There were relatively low proportions of women who could correctly answer about suspected symptoms, causes, curability and complications of STI. Urethral discharge in men, an important symptom of STI was neglected by the study respondents, and majority of the women did not report knowledge of any symptom of STI. Meanwhile, the concepts that bad hygiene, sex during menses or soon after delivery, multiple childbirths or abortions are "causes" of STI, and untreated STI can lead to HIV/AIDS also existed among the respondents. These findings are consistent with other qualitative studies in Vietnam showing limited knowledge [15,18] and misconceptions regarding STI among people in the community [15,19].

Our study showed a certain number of the study women did not know if STI can be prevented, and a low percentage of women gave correct answers about STI prevention. Condom use is considered the single, most efficient, available means to reduce the sexual transmission of both HIV and STI [23]. Nevertheless, in Vietnam, low knowledge of condom use for STI prevention among the general population has been previously shown [24]. A study in India also shows 22% of young girls do not know about condom use could protect people from STI [25]. Among our respondents however, especially among the unmarried women, this knowledge was even less. Whereas, studies show that 92% of young people in Ho Chi Minh city know that the use of condoms protects against HIV [26], and that there is existence of adequate knowledge of HIV but little concern for STI among female sex workers [20]. The difference could possibly be due to an unbalanced effort that has been given to the combat against HIV/AIDS since 97.5% of the government funds were allocated for HIV/AIDS prevention and care, while only 2.5% were for STI control activities [27]. This might put people at increased risk because of poor concern for STI. Besides, nearly half of our respondents either did not know the necessity of partner treatment or claimed that it was not necessary. Insufficient knowledge regarding STI prevention and partner treatment among the study women may result in neglecting the risks of unsafe sex.

Studies have shown that in a high-income society, better knowledge is not related to income or residence but related to higher education [28,29], having ever had sex [29], and STI history [28,30]. Being knowledgeable about RTI was related to higher probability of self-reported symptoms has been shown among rural Chinese women [31]. Our results showed that the overall STI knowledge was higher among married women and among those who had had self-reported symptoms during the past six months. Women with low education or low economic status had less knowledge of STI than those with higher education or economic category. Moreover, the results demonstrated an obvious association between low STI knowledge and residency in the mountainous and highland areas. This may reflect the fact that women with low education and from rural or remote areas wait before seeking care for STI [16]. Lack of awareness of STI consequences among our respondents may have led delayed treatment.

In our study, up to one-third of the unmarried women reported no knowledge about STI. Moreover, the women under the age of 20 demonstrated the lowest level of STI knowledge. This may be partially due to the sensitive nature of the issue and feelings of shame among women, especially unmarried women, when talking about STI [15,18].

To our knowledge, one new finding in our study is the impact of experience of induced abortion, but not of childbirth, on STI knowledge. Those who have experienced an induced abortion might have been provided information, by healthcare providers, about the prevention of unwanted pregnancies, which is associated with STI knowledge. However, women who go to health facilities for childbirth might not receive information about diseases related to sexuality. Thus, how healthcare providers phrase their information may play an important role in improving people's STI knowledge.

Furthermore, the analysis showed that the intra-cluster correlation coefficient was significantly greater than 0 (p < 0.01), which indicated the existence of relations between knowledge levels of women in each cluster. This may be caused by information acquired from peers/friends within the cluster. This could be easily understood by the fact that women having symptoms usually seek help or advice from peers/friends and healthcare providers [15]. Studies in Vietnam and elsewhere show that HIV/AIDS information is derived mainly from friends [32], healthcare providers [32], and mass media [20,24,32]. Therefore, peer/friend education, informal conversations between women within clusters, mass communication, and the use of healthcare providers as means of providing information to the community should be taken into consideration when designing and implementing intervention programmes.

### Methodological considerations

Since we had no drop-outs, one might question how we obtained such a high response rate. This was probably due to a number of different reasons. Firstly, we have had good cooperation with the households and the commitment of local authorities to the FilaBavi. Secondly, the interviewers were female surveyors of FilaBavi, who have been well-trained in doing household surveys and have created good relationships with households. The face-to-face interview can clarify questions, and usually get high response rates because the presence of the interviewer encourages participation and involvment of participants [33]. Thirdly, the purposes and procedures of the study were clearly explained before the data collection and the married women were eager to participate in the gynaecological examination to be performed by experienced female doctors from Hanoi together with some sophisticated tests and treatment provided free of charge [14]. Lastly, when designing the study and planning for data collection, we deliberately chose the most appropriate time of the year in order to avoid the harvest period and thus facilitate the participation of the study subjects.

According to the pilot study, it was too sensitive and impossible to include questions about respondents' and/or their husbands'/partners' sexual behaviours or risks for STI/HIV. Furthermore, the topic of this study is possibly sensitive for face-to-face interviews and people are reluctant to disclose their sexual behaviours and may be reluctant to express even their knowledge. Besides, respondents might felt that their answers were not anonymous since the surveyors lived in the area and were known to them and would also come back again for the routine data collection of FilaBavi, therefore, they might have been less informative or open. Consequently, our results possibly underestimated women's knowledge about STI, especially unmarried women's, and did not reflect the magnitude of risks that women and their husbands/partners are engaged in. These problems might have been less by using a self-administered questionnaire [33]. Because of the selection of only women, our results are presumably representative of the female part of the population in Bavi district and possibly other rural areas of Vietnam.

Concerning the scoring, the answers were not weighed since we assumed each item equally. Alternative regression models using weighed scores were also performed (results not presented); the outcomes however, did not differ. We considered the interviewer variances had no significant impact on the results as the intra interviewer correlation coefficient was small (ICC = 0.08).

## Conclusion

In conclusion, low levels of STI knowledge among the study respondents reflect the potential importance of health education interventions to improve STI knowledge for the general population. Young and unmarried women, the most vulnerable population should be specifically targeted. Integration of STI and HIV/AIDS into health education materials could be considered. Intervention programmes should be diversified and tailor-made for each group. The association between experience of induced abortion and higher level of STI knowledge implies the role of HCPs in health education.

## Competing interests

The authors declare that they have no competing interests.

## Authors' contributions

PTL designed the study, supervised the data collection, performed data analysis, and main writing. CSL, IM and NTKC supported in the study design and the data collection, HDP contributed to the data collection and statistical analysis. All authors read and approved the final manuscript.

## Pre-publication history

The pre-publication history for this paper can be accessed here:

http://www.biomedcentral.com/1471-2334/9/85/prepub

## Supplementary Material

Additional file 1 **Questionnaire**. the questionnaire contains questions regarding respondents' socio-demographic information; experiences related to childbearing; and questions concerning STI knowledge. Click here for file

## References

[B1] Mayaud P Mabey D Approaches to the control of sexually transmitted infections in developing countries: old problems and modern challenges Sex Transm Infect 2004 80 3 174 182 15169997 10.1136/sti.2002.004101 PMC1744836

[B2] Fleming DT Wasserheit JN From epidemiological synergy to public health policy and practice: the contribution of other sexually transmitted diseases to sexual transmission of HIV infection Sex Transm Infect 1999 75 1 3 17 10448335 10.1136/sti.75.1.3 PMC1758168

[B3] Wasserheit JN The significance and scope of reproductive tract infections among Third World women Int J Gynecol Obstet 1989 Suppl 3 145 168 10.1016/0020-7292(89)90115-X 2686703

[B4] WHO Global strategy for the prevention and control of sexually transmitted infections: 2006–2015. Breaking the chain of transmission 2007 Geneva: WHO

[B5] WHO Sexually transmitted and other reproductive tract infections: a guide to essential practice 2005 Geneva: WHO

[B6] Mmbaga EJ Leyna GH Mnyika KS Klepp KI Sexually transmitted infections knowledge and its impact in the practice of risky sexual behaviours and HIV serostatus: results from rural Kilimanjaro, Tanzania Sex Transm Infect 2008 84 3 224 226 10.1136/sti.2007.029488 18283095

[B7] Rekart ML Sex in the city: sexual behaviour, societal change, and STDs in Saigon Sex Transm Infect 2002 78 Suppl 1 i47 54 12083447 10.1136/sti.78.suppl_1.i47 PMC1765813

[B8] WHO Regional Office for the Western Pacific. Consensus report on STI, HIV and AIDS epidemiology: Vietnam http://www.wpro.who.int/NR/rdonlyres/5E7E8481-C40C-457F-BFBD-FC1D4F9583ED/0/Consensus_Report_VTN_2000.pdf2097487

[B9] Chalker J Chuc NT Falkenberg T Do NT Tomson G STD management by private pharmacies in Hanoi: practice and knowledge of drug sellers Sex Transm Infect 2000 76 4 299 302 11026888 10.1136/sti.76.4.299 PMC1744190

[B10] United Nations Summary of the HIV epidemic in Vietnam http://www.unaids.org.vn/facts/docs/key_messages_sep_2006_e.pdf

[B11] Nguyen VT Nguyen TL Nguyen DH Le TT Vo TT Cao TB O'Farrell N Sexually transmitted infections in female sex workers in five border provinces of Vietnam Sex Transm Dis 2005 32 9 550 556 10.1097/01.olq.0000175415.06716.6d 16118603

[B12] Thuong NV Nhung VT Nghia KV Tram LT O'Farrell N HIV in female sex workers in five border provinces of Vietnam Sex Transm Infect 2005 81 6 477 479 16326850 10.1136/sti.2005.016097 PMC1745073

[B13] Ministry of Health Results from the HIV/STI Integrated Biological and Behavioral Surveillence (IBBS) in Vietnam 2005–2006. Ministry of Health, Vietnam http://www.fhi.org/en/HIVAIDS/pub/survreports/res_IBBS_2005-6_Vietnam.htm

[B14] Lan PT Lundborg CS Phuc HD Sihavong A Unemo M Chuc NT Khang TH Mogren I Reproductive tract infections including sexually transmitted infections: a population-based study of women of reproductive age in a rural district of Vietnam Sex Transm Infect 2008 84 2 126 132 10.1136/sti.2007.027821 18003708

[B15] Lan PT Faxelid E Chuc NT Mogren I Lundborg CS Perceptions and attitudes in relation to reproductive tract infections including sexually transmitted infections in rural Vietnam: A qualitative study Health Policy 2008 86 2–3 308 317 10.1016/j.healthpol.2007.11.007 18191494

[B16] Thi Thu H Ziersch A Hart G Healthcare-seeking behaviours for sexually transmitted infections among women attending the National Institute of Dermatology and Venereology in Vietnam Sex Transm Infect 2007 83 5 406 410 10.1136/sti.2006.022079 17314126 PMC2659041

[B17] UNFPA Research on reproductive health in Vietnam. A review for the period 2000–2005 Hanoi, Vietnam 2007

[B18] Nguyen HN Liamputtong P Murphy G Knowledge of contraceptives and sexually transmitted diseases and contraceptive practices amongst young people in Ho Chi Minh City, Vietnam Health Care Women Int 2006 27 5 399 417 10.1080/07399330600629542 16877291

[B19] Binh NTH Gardner M Elias C Perceptions of morbidity related to reproductive tract infection among women in two rural communities of Ninh Binh Province, Vietnam Culture, Health & Sexuality 2002 4 153 171 10.1080/13691050110096170

[B20] Ngo AD Ratliff EA McCurdy SA Ross MW Markham C Pham HT Health-seeking behaviour for sexually transmitted infections and HIV testing among female sex workers in Vietnam AIDS Care 2007 19 7 878 887 10.1080/09540120601163078 17712691

[B21] Nguyen TKC Diwan VK FilaBavi, a demographic surveillance site, an epidemiological field laboratory in Vietnam Scandinavian Journal of Public Health 2003 31 3 7 10.1080/14034950310015031 14578073

[B22] Go VF Quan VM Chung A Zenilman J Hanh VT Celentano D Gender gaps, gender traps: sexual identity and vulnerability to sexually transmitted diseases among women in Vietnam Soc Sci Med 2002 55 3 467 481 10.1016/S0277-9536(01)00181-2 12144153

[B23] WHO Global strategy for the prevention and control of sexually transmitted infections: 2006–2015: breaking the chain of transmission 2007 Geneva: WHO

[B24] Bui TD Pham CK Pham TH Hoang LT Nguyen TV Vu TQ Detels R Cross-sectional study of sexual behaviour and knowledge about HIV among urban, rural, and minority residents in Viet Nam Bull World Health Organ 2001 79 1 15 21 11217661 PMC2566334

[B25] McManus A Dhar L Study of knowledge, perception and attitude of adolescent girls towards STIs/HIV, safer sex and sex education: (a cross sectional survey of urban adolescent school girls in South Delhi, India) BMC Women's Health 2008 8 12 18647417 10.1186/1472-6874-8-12 PMC2490677

[B26] Vinh DT Raguin GL Thebaud Y Semaille C Tri LD Knowledge, attitudes, belief and practice related to HIV/AIDS among young people in Ho Chi Minh City, Vietnam Eur J Epidemiol 2003 18 8 835 836 10.1023/A:1025346015455 12974561

[B27] Nguyen TH Nguyen TL Trinh QH HIV/AIDS epidemics in Vietnam: evolution and responses AIDS Educ Prev 2004 16 3 Suppl A 137 154 15262572 10.1521/aeap.16.3.5.137.35527

[B28] Grulich AE de Visser RO Smith AM Rissel CE Richters J Sex in Australia: knowledge about sexually transmissible infections and blood-borne viruses in a representative sample of adults Aust N Z J Public Health 2003 27 2 230 233 10.1111/j.1467-842X.2003.tb00813.x 14696716

[B29] Lim MS Hellard ME Aitken CK Hocking JS Sexual-risk behaviour, self-perceived risk and knowledge of sexually transmissible infections among young Australians attending a music festival Sex Health 2007 4 1 51 56 10.1071/SH06031 17382039

[B30] Andersson-Ellstrom A Milsom I Knowledge about the prevention of sexually transmitted diseases: a longitudinal study of young women from 16–23 years of age Sex Transm Infect 2002 78 5 339 341 12407234 10.1136/sti.78.5.339 PMC1744534

[B31] Xia DY Liao SS He QY Choi KH Mandel JS Self-reported symptoms of reproductive tract infections among rural women in Hainan, China: prevalence rates and risk factors Sex Transm Dis 2004 31 11 643 649 10.1097/01.olq.0000143111.33741.40 15502670

[B32] Raheel H White F Kadir MM Fatmi Z Knowledge and beliefs of adolescents regarding sexually transmitted infections and HIV/AIDS in a rural district in Pakistan J Pak Med Assoc 2007 57 1 8 11 17319411

[B33] Robson C The methods of data collection. In: Real World Research. A resource for social scientists and practitioner-researcher 2002 Second Cornwall: Blackwell Publishing Ltd 223 30712462445

