# Supplementary material for: Lack of knowledge about sexually transmitted infections among women in North rural Vietnam
Source: BMC Infect Dis. 2009 Jun 6;9:85. doi: 10.1186/1471-2334-9-85 (PMC2701953; doi:10.1186/1471-2334-9-85)
Supplement: Additional file 1 — Questionnaire. the questionnaire contains questions regarding respondents' socio-demographic information; experiences related to childbearing; and questions concerning STI knowledge. [file 1471-2334-9-85-S1.doc]

**Questionnaire on Sexually Transmitted Infections**

*For face-to-face interviewing women aged 15 to 49 in FilaBavi, Vietnam*

Date of interview:...../...../.....time….. Date of supervision:......./......./........

Name of interviewer:.............Duration…….. Name of field supervisor:.....................

Comments of interviewer on respondent’s Comments of field supervisor

cooperation……………………………… …………………………………………. ………………………………………… ………………………………………….

**I. General information:**

Commune................ Cluster number................ Household code................

Name ............................................... Age................ ID number................

Ethnic group: 1. Kinh 2. Muong 3. Other

**1. a, Your occupation:**

1. Farmer 2. Government staff 3. Worker

4. Trader 5. Student 6. Housewife

7. Unemployed 8. Hired labor

9. Service *(please specify*).......................................................................

10. Other *(please specify*).......................................................................................

**b,** Do you have to soak your body into water while working? 0. No 1. Yes

**2. Your education:**

1. Illiterate 2. Primary 3. Secondary school

4. High school 5. College or University

**3. a, Your marital status at present:**

1. Unmarried 2. Married, living with husband/partner

3. Separated 4. Divorced

5. Widowed

**b,** Year of your first marriage?………………

**4. Have you had any children?** 0. No 1. Yes**,** If yes, how many …………

5. Which of the following events have you ever experienced?

|  |  | No | Yes | How many times | When was the last time? |
| --- | --- | --- | --- | --- | --- |
| 1 | Induced abortion |  |  |  |  |
| 2 | Miscarriage |  |  |  |  |
| 3 | Still birth |  |  |  |  |
| 4 | Premature birth |  |  |  |  |
| 5 | Neonatal death |  |  |  |  |

**6. What contraceptive methods are you currently using?** *(can be many)*

1. Intrauterine device 2. Condom

3. Calendar/mucus method 4. Withdrawal

5. Contraceptive pill 6. Male sterilization

7. Female sterilization

8. Others *(please specify*).......................................................................

9. None

### II. Questions about STI knowledge

**7. a, What do you think about vaginal discharge in women?**

1. Normal 2. Abnormal 3. Don't know

1. Seldom 2. Common 3. Don't know

**b,** How would you define vaginal discharge as abnormal? *(can be many)*

1. Greater amount than usual 0. No 1. Yes

2. Odor 0. No 1. Yes

3. Yellow or green discharge 0. No 1. Yes

4. Powdery liquid 0. No 1. Yes

5. Foamy liquid 0. No 1. Yes

6. Blood-stained liquid 0. No 1. Yes

7. Don't know

8. Other *(please specify*)........................................................................................

**8*.* Do you know what are considered suspected symptoms of STI ?** *(can be many)*

1. Abnormal vaginal discharge (female)

2. Urethral discharge (male)

3. Genital ulcers 4. Genital warts

5. Genital itching 6. Pain during urination

7. Pain during sexual intercourse 8. Lower abdominal pain (female)

9. Don't know

10. Other *(please specify*).......................................................................

**9. Please tell what are possible “causes”** (based on respondents’ own words) **of STI?** *(can be many)*

1. Bacteria 2. Virus

3. Fungus 4. Bad hygiene of man

5. Bad hygiene of woman 6. Being unfaithful

7. Intrauterine device 8. Having sex soon after delivery

9. Sex during menses 10. Intravenous drug use

11. Blood transfusion 12. Multiple abortion/childbirths

13. Using unclean water 14. Soaking body in water

15. Other *(please specify*).......................................................................................

16. Don't know

**10. a, Can STI transmit?**

0. No 1. Yes 3. Don't know

**b,** If yes, what are routes of transmission? *(can be many)*

1. Sexual intercourse 2. Blood transfusion

3. Sharing needle 4. Mother to child

5. Sharing clothes, things 6. Don't know

7. Other *(please specify*).......................................................................

**11*.* Is it necessary to treat the husband/wife/partner of STI patients'?**

0. No 1. Yes 3. Don't know

**12. a, In your opinion, can STI be cured?**

0. No 1. Yes 3. Don't know

**b,** If yes, which ones *(please specify*)...........................................................................

**c,** If no, which ones *(please specify*).............................................................................

**13. What are complications of STI if untreated?** *(can be many)*

1. Infertility 2. Ectopic pregnancy 3. Cervic cancer

4. Premature birth 5. Still birth 6. Miscarriage

7. Neonatal death 8. Do not know

9. Other *(please specify*)........................................................................................

**14. Can STI be prevented?**

0. No 1. Yes 3. Don't know

*If yes, how? (please specify*)...................................................................................

........................................................................................................................................

**III. Questions about symptoms**

**15. a, Have you had any “inflammation”** (this is a common term used among people in the study site for reproductive tract infection**) or STI during your life?**

0. No 1. Yes

**b,** If yes, which disease/symptoms……………

**c,** If yes, how many times?..............time(s); when was the last episode?...............

**16.Have you had any of the following symptoms during the last 6 months?**

|  | 0. No | 1. Yes |
| --- | --- | --- |
| 1. Abnormal vaginal discharge |  |  |
| 2. Genital itching |  |  |
| 3. Dysuria (pain during urination) |  |  |
| 4. Dyspareunia (pain during intercourse) |  |  |
| 5. Bleeding after sexual intercourse |  |  |
| 6***.*** Warts in the vulva, vagina, anus |  |  |
| 7***.*** Ulcers in the vulva, vagina, anus |  |  |
| 8. Lower abdominal pain *(please specify if it relates to menstruation)* |  |  |
| 9. Bleeding between menses |  |  |
| 10. Menstruation extended in relation to previously |  |  |
| 11. Bleedings increased |  |  |
|  |  |  |

**17. Do you have any following symptoms at present?**

|  | 0. No | 1. Yes |
| --- | --- | --- |
| 1. Abnormal vaginal discharge |  |  |
| 2. Genital itching |  |  |
| 3. Dysuria (pain during urination) |  |  |
| 4. Dyspareunia (pain during intercourse) |  |  |
| 5. Bleeding after sexual intercourse |  |  |
| 6. Warts in the vulva, vagina, anus |  |  |
| 7. Ulcers in the vulva, vagina, anus |  |  |
| 8. Lower abdominal pain *(please specify if it relates to menstruation)* |  |  |
| 9. Bleeding between menses |  |  |
| 10. Menstruation extended in relation to previously |  |  |
| 11. Bleedings increased |  |  |

**Thank you very much for your answers!**
